# Effects of bubble cutting dynamic behaviors on microalgal growth in bubble column photobioreactor with a novel aeration device

**DOI:** 10.3389/fbioe.2023.1225187

**Published:** 2023-06-28

**Authors:** Sha Zhao, Wenyue Feng, Jinming Li, Xiaoguang Zhang, Li Liu, Hongyan Li

**Affiliations:** College of Electromechanical Engineering, Qingdao University of Science and Technology, Qingdao, China

**Keywords:** microalgae carbon sequestration, visualization, bubble cutting, aeration device, hydrophilicity, chlorella pyrenoidosa

## Abstract

**Introduction:** Carbon sequestration by microalgae is an effective approach for achieving carbon neutrality owing to its high carbon capture efficiency and environmental friendliness. To improve microalgae CO_2_ fixation efficiency, various methods to enhance CO_2_ transfer at the gas-liquid interface have resulted in high energy consumption.

**Methods:** In this study, a novel aeration device with bubble cutting slices was installed in a photobioreactor for CO_2_ supply, which could precisely separate bubbles into sizes on the way to rising after departure, achieving CO_2_ transfer enhancement without extra energy consumption. Subsequently, the bubble cutting dynamic behaviors in the photobioreactor were studied, and the effects of thickness, hydrophilicity, and arrangement of cutting slices on microalgal growth were analyzed.

**Results:** It was found that bubble cutting caused the maximum dry weight and biomass productivity of microalgae to improve by 6.99% and 33.33%, respectively, compared with those of the bioreactor without cutting units, owing to a 27.97% and 46.88% decrease in bubble size and rising velocity, respectively, and an 84.55% prolongation of bubble residence time.

**Discussion:** Parallel cut slices with a thickness and spacing of less than 3 mm successfully cut the bubbles. The hydrophobic slice surface prevented daughter bubble departure and prolonged the bubble residence time, impeding microalgae growth owing to bubble coalescence with subsequent bubbles. The optimal cutting slice parameters and culture conditions for microalgal growth were 1 mm slice thickness, less than 1 mm slice spacing, 5% inlet CO_2_ concentration, and 70 mL/min gas flow rate.

## 1 Introduction

With the rapid development of economy CO_2_ emissions, mushrooming has increased atmospheric CO_2_ concentration by 50% since the industrial revolution, increasing mean annual global temperatures to 0.87°C (Kumar et al., 2018; Ruiz-Ruiz et al., 2020). Many adverse environmental effects threaten human survival, including climate warming, sea level rise, and melting glaciers (Meinshausen et al., 2009; House et al., 2011). Faced with such critical challenges, over two-thirds of the countries and regions aim to achieve carbon neutrality between 2030 and 2070 to reduce CO_2_ emissions (Jin et al., 2023; Kong et al., 2023; Xu et al., 2023). Therefore, developing effective and sustainable carbon capture, utilization, and storage technologies is becoming increasingly urgent, and much effort has been made for several decades by researchers (Grimston et al., 2001; Diao et al., 2004; Seth and Wangikar, 2015). Among the various carbon capture, utilization, and storage technologies, microalgal carbon sequestration is considered an effective approach to achieving carbon neutrality owing to its high carbon-capture efficiency, many valuable products, and environmental friendliness (Ugwu et al., 2008; Yang et al., 2014; Chang et al., 2023). Microalgal carbon sequestration is always conducted in a bubble column photobioreactor where CO_2_ mixed gas enters the microalgae suspension as bubbles. As the mixed gas bubbles rise, the CO_2_ molecules in the bubbles are transported across the gas-liquid interface, dissolved into the microalgae suspension, and bio-fixed by microalgae photosynthesis. During the entire CO_2_ transfer process, the maximum mass transfer resistance occurs at the gas-liquid interface (McGinn et al., 2011; Raeesossadati et al., 2014). Thus, CO_2_ transfer and dissolution at the gas-liquid interface directly affect microalgal growth and CO_2_ fixation efficiency (Wongsuchoto et al., 2003; Bitog et al., 2011). Although various CO_2_ transfer enhancement approaches have been used at the gas-liquid interface, such as microbubble generation, ultrasonication, and stirring, they result in high energy consumption. Adding cutting slices on the way of bubble rising can separate bubbles into different sizes without affecting bubble formation and departure processes, promoting CO_2_ mass transfer and fixation by extending the gas-liquid interface area, prolonging the bubble residence time, and strengthening the interface disturbance without extra energy consumption. The increase in the surface and wave energies at the gas-liquid interface is primarily due to decreased kinetic energy during the bubble rise. Therefore, applying bubble cutting to the optimal design of an aeration device should significantly improve the performance of the bubble column photobioreactor. Furthermore, the effects of bubble cutting behavior require in-depth discussion.

Research on CO_2_ dissolution and transportation enhancement at gas-liquid interfaces in microalgae photobioreactors have been conducted for several decades (Ding et al., 2016) and has primarily focused on producing small bubbles, strengthening interface fluctuation, and prolonging bubble residence time. To improve the CO_2_ dissolution rate, the production of small bubbles is considered an effective method owing to the enlargement of the gas-liquid contact area. Therefore, Falinski et al. (2018) used a fine pore air stone as the aerator to generate small bubbles with diameters of 0.5–2.0 mm, which significantly improved the mass transfer coefficient of CO_2_ to 573.2 h^−1^. Similarly, to further decrease bubble size, Cheng et al. (2019) used a new microporous fibrous-diaphragm aerator (3D printing technology) with an average pore diameter of 28 μm, which creates a microbubble diameter of 0.91 mm, increasing CO_2_ mass transfer coefficient by 40%. Furthermore, a hollow fiber membrane was adopted by Zheng et al. (2016) to promote CO_2_ mass transfer; however, it could not be used because it was easily blocked by algae. However, all enhancement methods above concentrated on the bubble formation phase and involved infinitely reducing the pore size to produce microbubbles, causing high breakthrough pressure to form microbubbles and gas pump operating costs. Although ultrasonication (Wu et al., 2021a; Wu et al., 2021b; Qin et al., 2022), stirring (Cheng et al., 2020; Naira et al., 2020; Wang et al., 2021a), and turbulence (Zhang et al., 2020; Wang et al., 2021b; Reichmann et al., 2021) can improve the mass transfer coefficient at the gas-liquid interface during bubble rising by reducing bubble sizes and strengthening the interface disturbance, they involved high energy consumption. Adding surfactants can produce small bubbles without additional energy consumption; however, surfactants have recycling complications (Li and Kang, 2020). Obstructing the bubble rise (Cheng et al., 2016; Yang et al., 2016; Xia et al., 2018; Ye et al., 2018) can lengthen its rising pathway, decrease its rising velocity, and prolong its residence time, conducive to CO_2_ dissolution and fixation. Baltussen et al. (2017) analyzed bubble cutting behaviors by installing a wire mesh on the bubble-rising path that could realize both bubble size reduction, residence time prolongation, and interface disturbance without extra energy consumption. However, bubbles reunited, and cutting failure always occurred owing to the small circular shape in the wire section and large three-phase contact interface resistance at the crossing point of the wire mesh. In this study, parallel cutting slices were installed in a novel aeration device in a photobioreactor to improve the bubble cutting success rate. The bubble cutting dynamic behaviors in the photobioreactor with the novel aerator were studied, and the effects of the thickness, arrangement, and hydrophilicity of the cutting slices on microalgal growth and CO_2_ fixation efficiency were analyzed.

## 2 Materials and methods

### 2.1 Microalgae and culture medium

The *Chlorella pyrenoidosa* employed in this study was purchased from the Institute of Hydrobiology, Chinese Academy of Sciences (Wuhan, China). *Chlorella pyrenoidosa* was cultivated in the BG11 medium (Xia et al., 2018).

### 2.2 Experimental system

**FIGURE 1:**
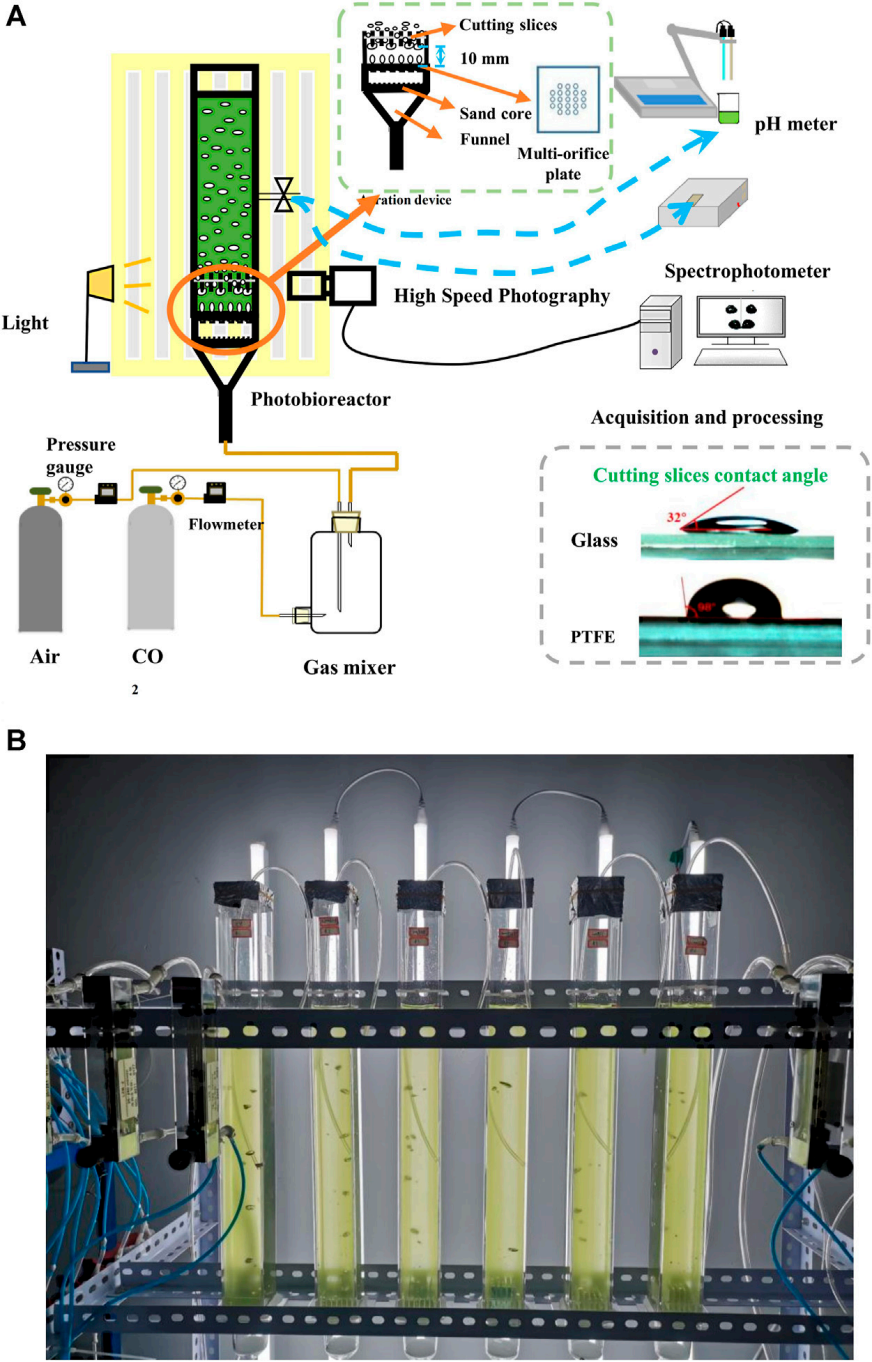
Effects of bubble cutting dynamic behaviors on microalgal growth in bubble column photobioreactor with a novel aeration device **(A)** Schematic of the experimental setup **(B)** Picture of the experimental setup.

Figure 1 shows the setup for studying the effects of bubble cutting dynamic behaviors on microalgal growth in a bubble column photobioreactor with a novel aeration device. All trials were conducted at 25°C. The illumination on the surface of the photobioreactors was maintained at 172 μmol/m^2^·s using fluorescent lamps placed parallel behind the photobioreactor. The photobioreactor comprised a transparent square tube and a novel aeration device fixed at the bottom of the square tube for CO_2_ gas supply. The inner cavity size of the square tube was 44 mm × 44 mm × 500 mm (length × width × height), with a working volume of 700 mL. The novel aeration device comprised a sand-core funnel, a multi-orifice plate, and cutting slices. The purchased sand core funnel was a glass funnel with a sand core pore size of 30–50 μm that allows for uniform gas flow. A multi-orifice plate was fixed on top of the sand core funnel to form bubbles with hole diameters and spacings of 1 and 6 mm, respectively. After detachment from the orifice, the bubbles rose straight before gradually transforming into a zigzag (Huang et al., 2017). Thus, the straight-rising stage was more suitable for bubble cutting.

To accurately cut bubbles without affecting bubble formation, cutting slices were installed in parallel, 10 mm above the center of the holes of the multi-orifice plate, which was placed on a fixed trestle attached to the top of the multi-orifice plate. The installation error should be kept within 1 mm. Pure CO_2_ (99.99%) and dry air supplied by gas cylinders were mixed thoroughly at different gas flow rates in a gas mixer (Ding et al., 2016) to obtain a mixed gas with the required CO_2_ concentrations. The mixed gas was injected from the bottom of the photobioreactor through a sand-core funnel and multi-orifice plate to form a uniform bubble flow. On contacting the cutting slices, the rising bubbles were blocked by the cutting slices due to surface tension and stretched by buoyancy on both sides of the cutting slices. After several times of deformation, bubbles were broken into small sizes, and the CO_2_ in the bubbles diffused, dissolved into the suspension, and was bio-fixed by microalgae. Two materials, glass and polytetrafluoroethylene (PTFE), with static contact angles of 32°and 98°, respectively, were used for the cutting slices. The pure glass slices were marked as gg, pure PTFE slices were marked as pp, and the combined slices obtained by pasting half glass and half PTFE were marked as gp. The height and length of the cutting slices were 24 and 38 mm, respectively, and the thickness was 0.15–3 mm.

### 2.3 Measurement method and performance assessment

The bubble cutting dynamic behaviors in the photobioreactor were recorded using high-speed photography (FASTCAM Mini UX100 at a shooting speed of 4,000 fps) with cold light as the backlight. The acquired bubble images were processed and analyzed using matrix laboratory (MATLAB) software. The images underwent grayscale conversion, interference removal, and bubble edge extraction using the Canny operator (Zhu et al., 2014). Subsequently, the bubble equivalent diameter and rising velocity were calculated. To better reflect the bubble behavior, more than one hundred bubbles in the area of height less than 82 mm in the photobioreactor were statistically analyzed owing to their more drastic bubble behavior variation. To facilitate the analysis, the area near the cutting slices from the square tube bottom (0–82 mm height) was divided into four parts in the height direction and marked as A_1_, A_2_, A_3,_ and A_4_. A_1_ (0–10 mm height) reflected the bubble behavior before cutting, and the average bubble diameter $\bar{d}$ was marked at 5 mm height. At A_2_ (height of 10–34 mm), the bubbles contacted the cutting slices and completed a severe deformation and cutting process. However, the equivalent bubble diameters were not calculated during cutting as they inaccurately reflected the bubble size. A_3_ (34–58 mm height) and A_4_ (58–82 mm height) reflected bubble-rising behaviors after cutting, and the average bubble diameter $\bar{d}$ was marked at heights of 46 and 70 mm, respectively. The bubble diameter and rising velocity listed in Table 1 adopted the average values in A_4_ owing to fewer changes in bubble behavior when the height was above 82 mm.

**TABLE 1:** Assessment of bubble behaviors and microalgal growth with different cutting units.

*δ* (mm)	*M* (mm)	Slice	Residence time (s)	Bubble diameter (mm)	Rising velocity (m/s)	*D*_max_ (g/L)	*P*_max_ (g/(L·d))
0	0	—	1.10 ± 0.048	5.97 ± 0.18	0.32 ± 0.011	1.43 ± 0.0099	0.18 ± 0.0087
0.15	0	gg	1.60 ± 0.046	4.72 ± 0.16	0.21 ± 0.0085	1.49 ± 0.0028	0.19 ± 0.0012
1	0	gg	2.03 ± 0.051	4.30 ± 0.22	0.17 ± 0.0115	1.53 ± 0.0086	0.24 ± 0.0093
3	0	gg	1.56 ± 0.040	6.75 ± 0.19	0.36 ± 0.016	1.37 ± 0.0065	0.17 ± 0.0084
1	0	gp	1.61 ± 0.045	5.04 ± 0.19	0.24 ± 0.01	1.43 ± 0.0075	0.19 ± 0.0092
1	0	pp	1.70 ± 0.039	4.76 ± 0.20	0.22 ± 0.011	1.48 ± 0.0067	0.20 ± 0.0046
0.15	1	gg	1.80 ± 0.040	4.86 ± 0.17	0.23 ± 0.0105	1.50 ± 0.0098	0.19 ± 0.1023
0.15	3	gg	1.26 ± 0.048	5.92 ± 0.20	0.31 ± 0.0126	1.31 ± 0.0087	0.16 ± 0.0029

Microalgal suspension samples were taken from the sampling connection of the photobioreactors every 12 h for testing. The hydrogen power (pH) was measured using a pH meter (Thermo Orion, United States). The microalgae concentration was represented by *D* (g/L dry weight of microalgae cells), calculated from the optical density measured using a spectrophotometer (TU 1901, China) when the wavelength was adjusted to 680 nm (*OD*_*680nm*_). The calculation formula for *Chlorella pyrenoidosa* dry weight was as follows (Ding et al., 2016):

$$D=0.51OD_{680nm}-0.029\left(R^{2}=0.99\right)$$ (1)

Microalgae biomass productivity *P* (g/(Ld)) represents the rapidity of microalgal growth, calculated using the following equation (Ding et al., 2016):

$$P=\frac{D^{\tau }-D^{0}}{\tau }$$ (2)

where $D^{\tau }$ (g/L) was the dry weight of microalgae on the day of *τ*, $D^{0}$ (g/L) was the initial dry weight of microalgae.

All studies were performed in duplicate. All measurements were repeated five times, and the data were presented as the average value and standard deviation.

## 3 Results and discussion

### 3.1 Effects of bubble behaviors on microalgal growth with cutting units in bioreactor

**FIGURE 2:**
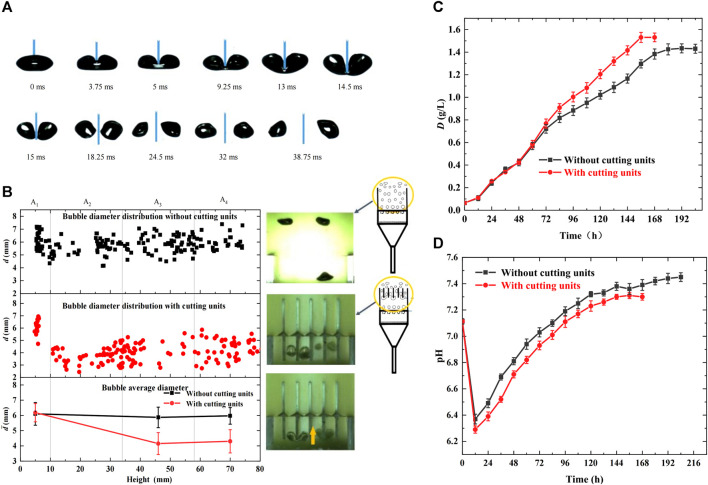
Effects of bubble behaviors on microalgal growth with cutting units in the bioreactor (cutting units:*δ* = 1 mm, *M* = 0, gg) **(A)** Bubble deformation process during bubble cutting **(B)** Distribution of bubble diameter in the height direction of bioreactor **(C)** Growth curve of *Chlorella pyrenoidosa* **(D)** Variation of pH during algal growth.

In this study, bubble dynamic behaviors were observed and compared in the photobioreactors with and without cutting units, and their effects on microalgal growth were analyzed. The cutting units used were glass slices of 1 mm thickness. The inlet CO_2_ concentration in the mixed gas was 5%, and the gas flow rate was maintained at 70 mL/min. The results are shown in Figure 2. Figure 2A shows the entire bubble deformation process during bubble cutting. The bubbles accelerated vertically in the morph of ellipsoid owing to buoyancy, inertial forces, surface tension, and fluid shear stress after departure from the orifices. On touching the bottoms of the glass slices, the bubble-rising velocity decreased. They deformed into a cashew morph, and the gas-liquid interface fluctuated significantly. Furthermore, the bubbles were stretched by buoyancy on both sides of the cutting slices, and middle necking emerged. Once the middle neck of the mother bubble broke, it was divided into two daughter bubbles. Therefore, the bubble cutting behaviors reduced the bubble size, enhanced the gas-liquid interface area and fluctuation, slowed the bubble rise, and prolonged the bubble residence time, conducive to CO_2_ dissolution and fixation. Figure 2B shows the bubble diameter distribution in the height direction of the bioreactor. Bubble diameters remained at approximately 6 mm in the entire bioreactor without cutting units, whereas an evident decrease was observed at 10 mm height owing to bubble cutting and were uniformly kept at approximately 4.3 mm during the subsequent rising process in the bioreactor with cutting units. Moreover, the statistical values in Table 1 reveal that adding cutting units decreased the bubble diameter by 27.97%, decreased the bubble rising velocity by 46.88%, and increased the residence time by 84.55%, all contributing to CO_2_ transfer and microalgal growth.

In addition, Figures 2C, D show the variation in dry weight and pH during algal growth, validating the above analysis. As shown in Figure 2C, in the first 60 h of growth, little differences were observed between the bioreactors with and without cutting units owing to the low algal cell concentration in suspension and lower CO_2_ demand. However, as microalgae grew, CO_2_ demand increased. The photobioreactor with cutting units had a greater growth rate and shorter growth time, and the maximum dry weight and biomass productivity reached 1.53 g/L and 0.24 g/(Ld), respectively, which were improved by 6.99% and 33.33%, respectively, compared with the bioreactor without cutting units. This is because the photobioreactor with cutting units had higher CO_2_ mass transfer and dissolution efficiency, promoting microalgal growth. Similarly, Figure 2D shows that the bioreactor with cutting units had lower pH during the entire growth process. Although bubble cutting may not achieve the equal increase of biomass productivity as existing enhancement technologies (Cheng et al., 2016; Yang et al., 2016; Xia et al., 2018; Ye et al., 2018), it causes lower energy consumption, longer bubble residence time, and bubble breakage accuracy.

### 3.2 Effects of bubble cutting unit thickness in aeration device

**FIGURE 3:**
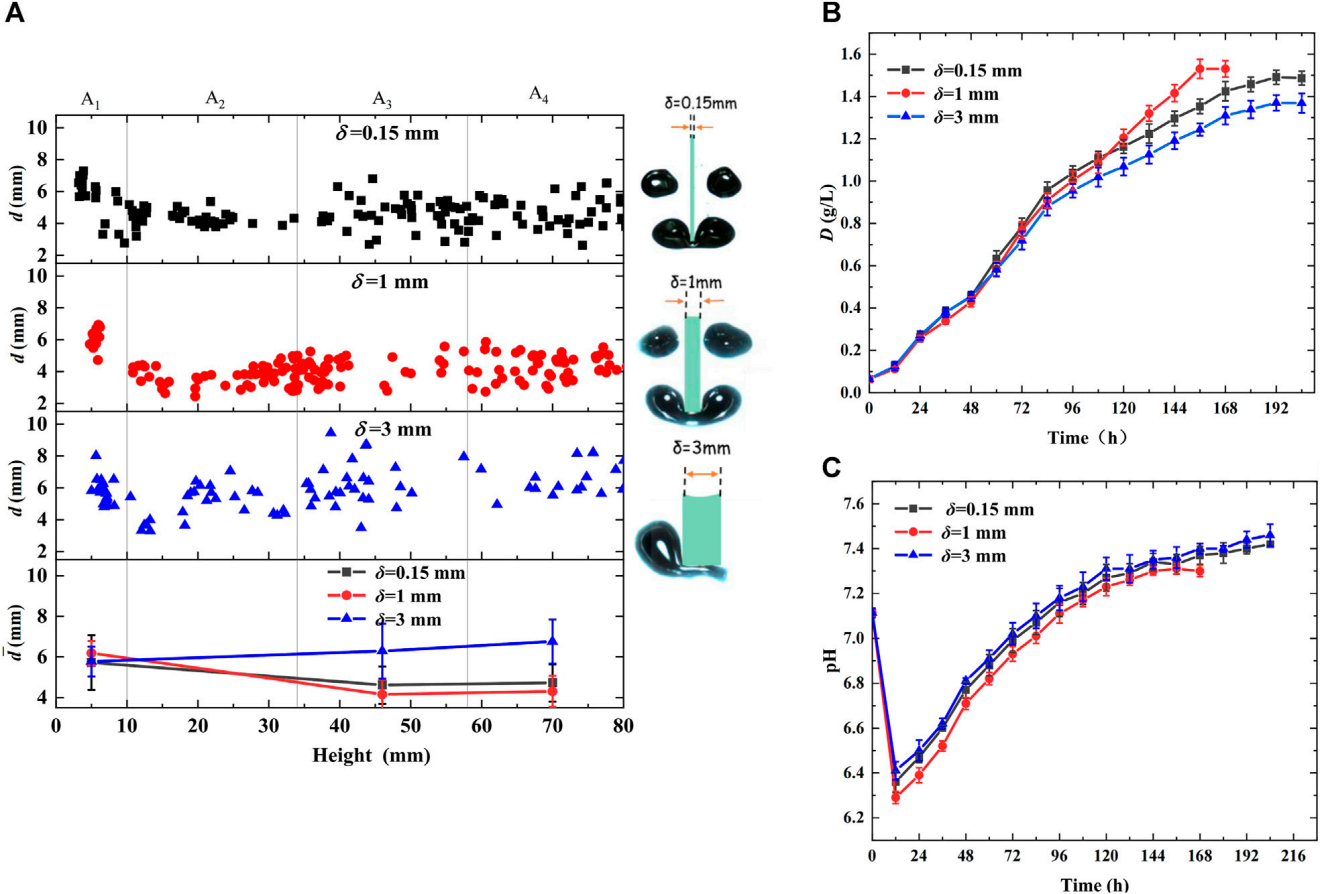
Effects of cutting units thickness on bubble behaviors and microalgal growth (cutting units: *M* = 0, gg) **(A)** Distribution of bubble diameter in the height direction of bioreactor **(B)** Growth curve of *Chlorella pyrenoidosa* **(C)** Variation of pH during algal growth.

To further optimize the cutting slice parameters, the effects of the glass cutting unit thickness (*δ* = 0.15, 1, and 3 mm) on bubble behavior and microalgal growth were investigated in the photobioreactor when the inlet CO_2_ concentration in the mixed gas was 5%, and the gas flow rate was controlled at 70 mL/min. The results are shown in Figure 3. Figure 3A shows the distribution of the bubble diameters in the height direction of the bioreactor. The bubble diameters decreased at 10 mm height when the cutting unit thickness *δ* was less than 1 mm owing to successful bubble cutting. The bubble diameters were uniformly at a low level of 4.72 and 4.3 mm when cutting unit thicknesses *δ* were 0.15 and 1 mm, respectively. At 3 mm slice thickness *δ*, the average bubble diameter remained larger than 6 mm and increased to 6.75 mm in A_4_. This is because the large thickness *δ* hindered the bubbles from forming a cashew morph owing to too small bubble heads on both sides of the cutting slices to provide buoyancy, causing bubbles to alternately shrink and stretch several times and slip away on one side of the slices owing to force imbalance, prolonging bubble residence time twice at A_2_ compared with that of a slice thickness *δ* of 1 mm, and increasing bubble size owing to coalescence with subsequent small bubbles. In addition, it can be deduced from the statistical values in Table 1 that a slice thickness *δ* of less than 1 mm decreased bubble size and rising velocity and prolonged the residence time, facilitating CO_2_ transfer and microalgal growth. At a thickness *δ* of 3 mm, although the residence time was prolonged by 41.82%, the bubble diameter and rising velocity increased by 13.07% and 12.50%, respectively, compared with those of the bioreactor without cutting units, which were adverse to CO_2_ dissolution and fixation.

Figures 3B, C show the variation in dry weight and pH during algal growth, reaching a similar conclusion to that of the above analysis. As shown in Figure 3B, in the primary growth stage, microalgae in the bioreactor with 1 mm thickness *δ* had the smallest growth rate owing to the low pH (Figure 3C) inhibiting microalgal growth, while the algal growth rate gradually increased to the highest as the microalgae grew, and the largest maximum dry weight and biomass productivity were achieved owing to the increased CO_2_ demand. For the photobioreactor with 3 mm thickness *δ*, the maximum dry weight and biomass productivity were reduced by 4.20% and 5.56%, respectively, compared with that of the bioreactor without cutting units. This is because the photobioreactor with 3 mm thickness *δ* had the lowest CO_2_ mass transfer and dissolution efficiency, impeding microalgal growth. Additionally, Figure 3C shows that the bioreactor with 3 mm thickness *δ* had the highest pH, while that with 1 mm had the lowest pH during the entire growth process is conclusive.

### 3.3 Effects of bubble cutting unit hydrophilicity in aeration device

**FIGURE 4:**
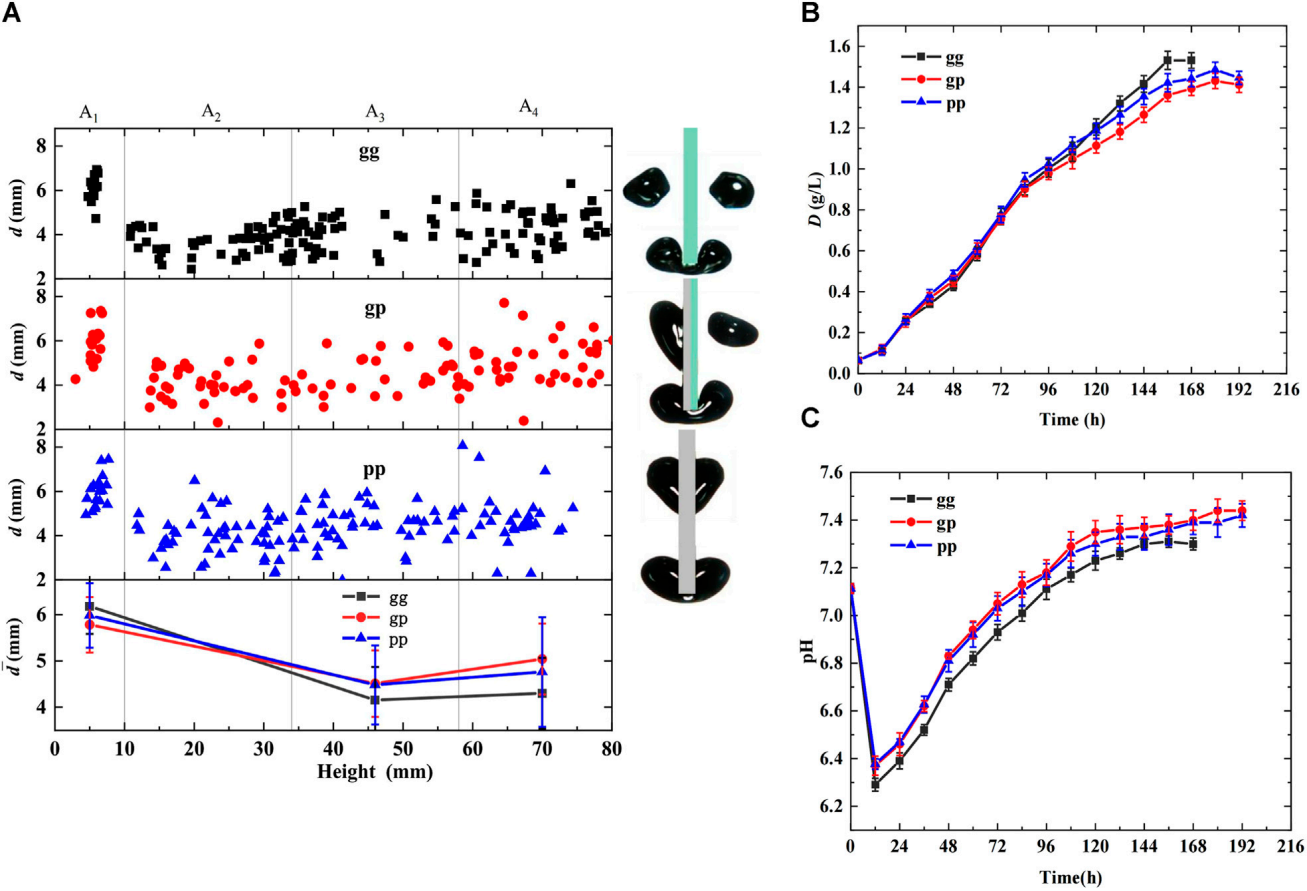
Effects of cutting units hydrophilicity on bubble behaviors and microalgal growth (cutting units:*δ* = 1 mm, *M* = 0) **(A)** Distribution of bubble diameter in the height direction of the bioreactor **(B)** Growth curve of *Chlorella pyrenoidosa* **(C)** Variation of pH during algal growth.

Hydrophobic slice surfaces may prevent daughter bubble departure and prolong bubble residence time, promoting CO_2_ and algal growth. To prove this hypothesis, the effects of the cutting unit hydrophilicity (gg, gp, pp) on bubble behavior and microalgal growth were investigated in a photobioreactor when the inlet CO_2_ concentration in the mixed gas was 5%, and the gas flow rate was maintained at 70 mL/min. The results are shown in Figure 4, where gg indicates that the cutting units were glass slices, pp indicates that the cutting units were PTFE slices, and gp indicates that the cutting units were combined glass and PTFE slices. Figure 4A shows the bubble diameter distribution in the height direction of the bioreactor. It can be concluded that the bubbles can be successfully cut in all three cases owing to the evident decrease in the bubble diameter at 10 mm height. The most uniform bubble diameter distribution and smallest average diameter were achieved when the cutting units were gg slices. The opposite results were obtained when the cutting units used gp. This was because the glass surfaces were hydrophilic and adversely affected the bubble attachment, whereas the PTFE surfaces were hydrophobic and conducive to bubble attachment. Thus, daughter bubbles struggled to depart from the PTFE cutting surfaces owing to the high surface tension and rose along cutting slices after bubble cutting, prolonging the bubble resistance time and causing coalescence with subsequent bubbles.

Regarding gp, the bubbles were cut quickly and unevenly owing to unbalanced forces, and the daughter bubbles at the PTFE surfaces always had larger diameters owing to the higher surface tension and bubble coalescence. A non-uniform bubble diameter distribution and the largest average diameter were obtained when the cutting units were gp slices. Furthermore, the statistical values in Table 1 show that smaller bubble sizes, lower rising velocities, and longer residence times were achieved using gg slices as the cutting unit, facilitating CO_2_ dissolution. In contrast, when gp was applied, the lowest decreases in bubble diameter and rising velocity were obtained at 15.58% and 25%, respectively, and the shortest residence time prolongation was 46.39% compared with those of the bioreactor without cutting units.

The growth curve and pH values shown in Figures 4B, C support the above analysis. Less distinct growth curves are shown in Figure 4B during the primary growth stage, and the bioreactor with gg slices had the lowest growth rate owing to its lower pH (Figure 4C). While evident differences occurred after 72 h of growth, the gg growth rate gradually increased to the highest and achieved the largest maximum dry weight and biomass productivity with an increased CO_2_ demand, owing to the highest CO_2_ mass transfer and dissolution efficiency. The maximum dry weight and biomass productivity of the gg slice were 7% and 26.32% higher than those of the gp slices, respectively, illustrating the inaccuracy of the initial assumption.

### 3.4 Effects of bubble cutting unit arrangement in aeration device

**FIGURE 5:**
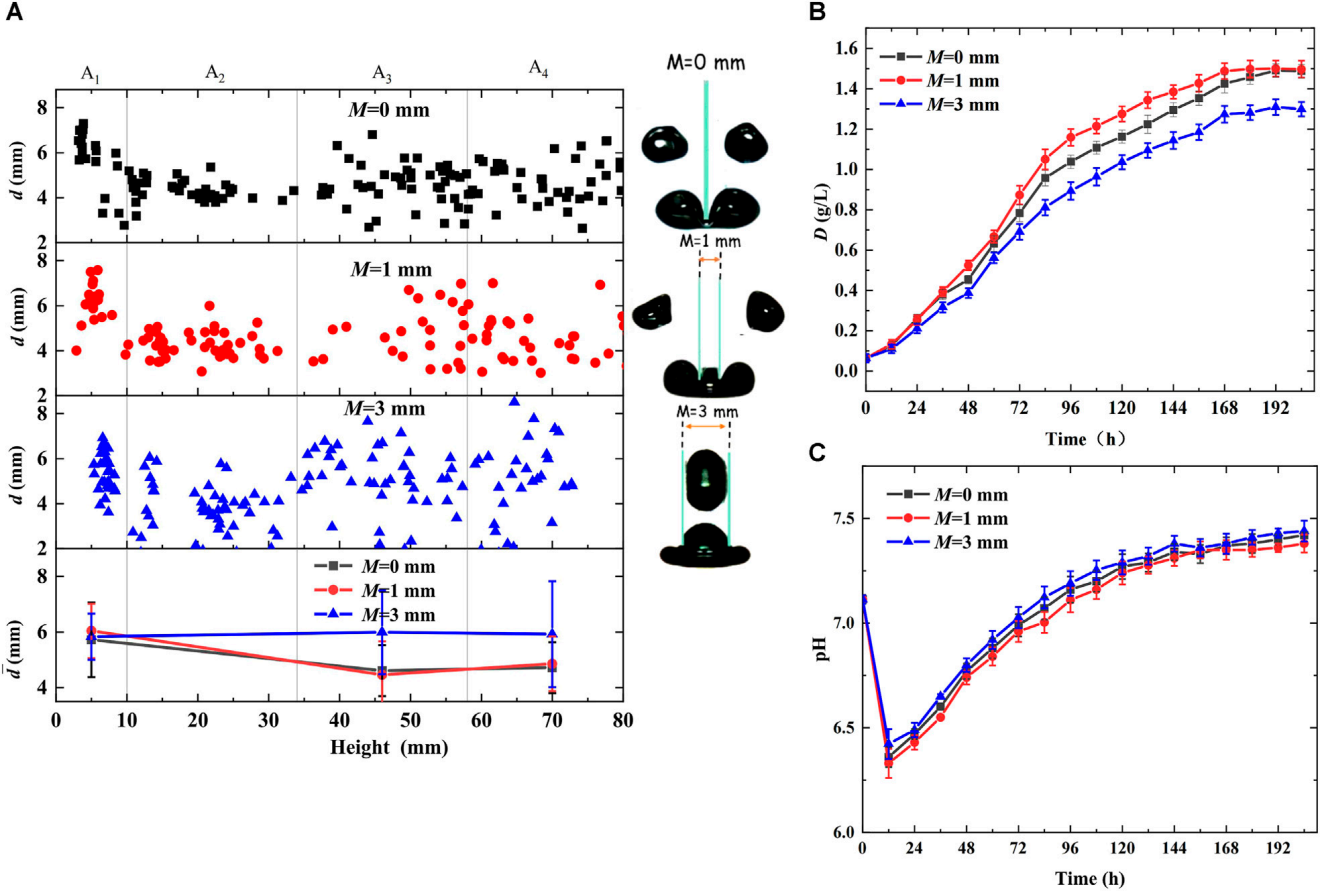
Effects of cutting units arrangement on bubble behaviors and microalgal growth (cutting units:*δ* = 0.15 mm, gg) **(A)** Distribution of bubble diameter in the height direction of the bioreactor **(B)** Growth curve of *Chlorella pyrenoidosa* **(C)** Variation of pH during algal growth.

To cut bubbles into smaller units, the effects of cutting unit arrangement on bubble behavior and microalgal growth were investigated in a photobioreactor. The cutting units used were glass slices of 0.15 mm thickness. The inlet CO_2_ concentration in the mixed gas was 5%, and the gas flow rate was maintained at 70 mL/min. Bubble cutting was challenging when the cutting slices were installed in crosses owing to the large three-phase contact interface resistance at the crossing. The bubbles coalesced and accumulated under the cutting units, and detached in a large size. All cut slices were arranged in parallel to improve the success rate of bubble cutting. Two cutting slices were installed above each row of holes, and the centerline of each row of holes was kept in the middle of the two cutting slices. Figure 5 shows the effects of the spacing between the two cutting slices (*M* = 0, 1, and 3 mm) on bubble behavior and microalgal growth. As shown in Figure 5A, the mother bubble was barely cut into three daughter bubbles. When the cutting slice spacings *M* were 0 and 1 mm, the bubbles could be cut into two parts, conclusive from the decreased bubble diameters at 10 mm height. When the cutting slice spacing *M* was increased to 3 mm, the average bubble diameter remained approximately 6 mm in region A_4_. This is because when the cutting slice spacing *M* is larger than 0 mm, bubbles form three heads when touching the cutting slices and oscillate owing to the imbalanced force, which can promote interface transportation and prolong the bubble residence time. A small spacing (*M* = 1 mm) caused a small middle bubble head and larger side heads, and the bubbles were easily separated into two parts owing to the larger buoyancy provided by the two side heads, causing the middle head to shrink. When *M* was increased to 3 mm, the middle bubble head became larger than the side heads, and the bubbles shrank thoroughly into spaces owing to the larger buoyancy in the middle. When the spacing *M* was between 1 and 3 mm, the bubble behaviors varied; they may be cut, shrink into spacing, or slip away owing to severe oscillation. Moreover, the statistical values in Table 1 reveal that a spacing *M* less than 1 mm decreased the bubble size and rising velocity and prolonged the residence time, facilitating CO_2_ transfer and microalgal growth. However, at 3 mm spacing *M*, the residence time was prolonged by 14.55%, and the bubble diameter and rising velocity were almost the same as those of the bioreactor without cutting units, which could not promote CO_2_ dissolution and fixation.

Figures 5B, C show the variation in dry weight and pH during algal growth, consistent with the above analysis. As shown in Figure 5B, at the beginning of 24 h growth, microalgae in the bioreactor with *M* of 0 and 1 mm had similar growth rates, whereas the algal growth rate gradually increased to the highest when *M* was 1 mm, and the largest maximum dry weight and biomass productivity were achieved owing to greater interface fluctuation and longer bubble residence time, facilitating CO_2_ transfer. However, the lowest maximum dry weight and biomass productivity were achieved in the photobioreactor with a 3 mm spacing *M* owing to the larger bubble size and shorter bubble residence time inhibiting CO_2_ mass transfer and microalgae growth. In addition, it is conclusive from Figure 5C that the bioreactor with a 3 mm spacing *M* had the highest pH, while that of 1 mm spacing *M* had the lowest pH during the entire growth process.

### 3.5 Performance of the optimal aeration device in microalgae photobioreactor

**FIGURE 6:**
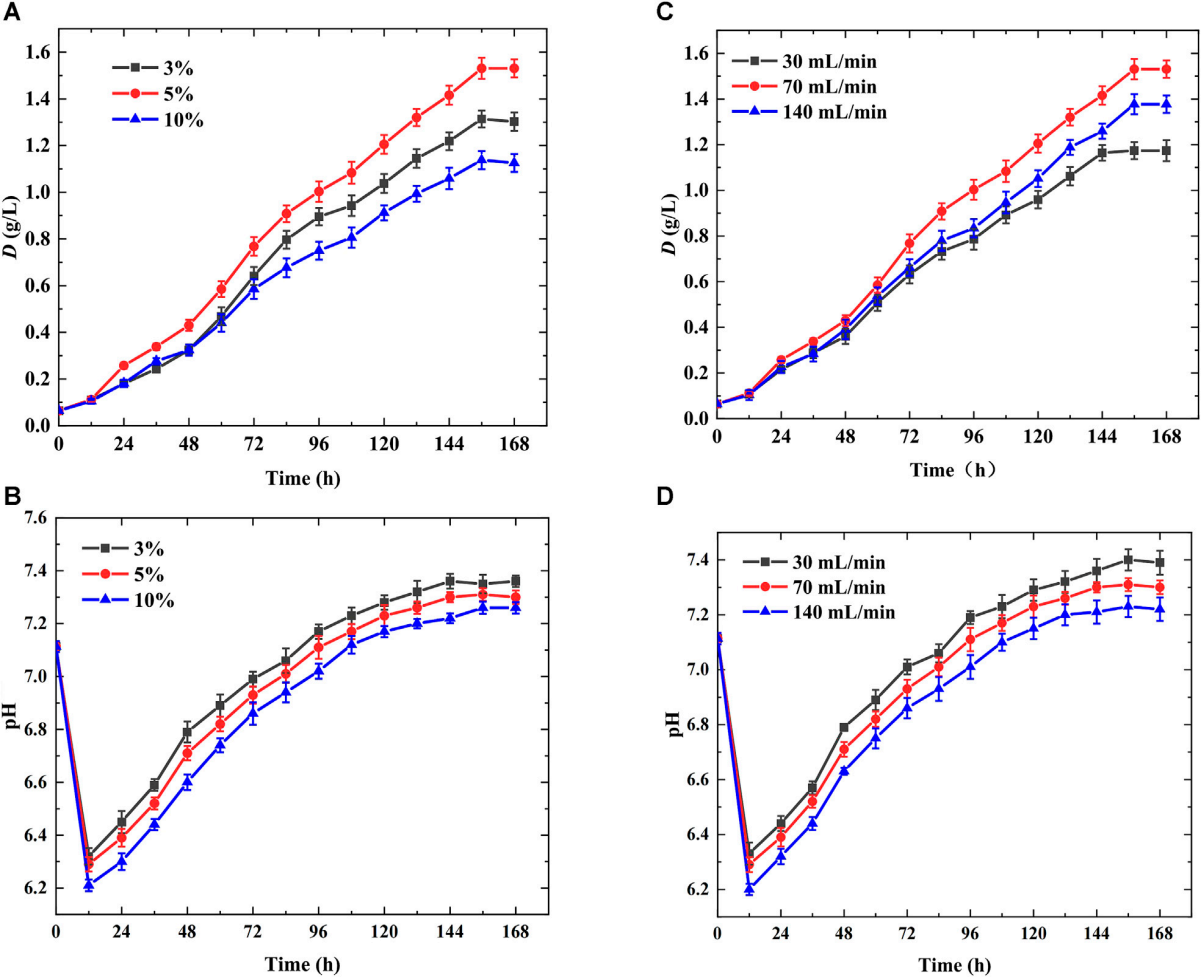
Performance of the optimal aeration device in microalgae photobioreactor (cutting units: *δ* = 1 mm, *M* = 0 mm, gg) **(A)** Variation of growth curve with different inlet CO_2_ concentrations **(B)** Variation of pH with different inlet CO_2_ concentrations **(C)** Variation of growth curve with different gas flow rates **(D)** Variation of pH with different gas flow rates.

The above studies indicated that bubble column photobioreactor with glass cutting slices (gg, *δ* = 1 mm, *M* ≤ 1 mm) parallelly arranged in the novel aeration device had the largest maximum dry weight and biomass productivity owing to smaller bubble size, lower rising velocity, and longer bubble residence time, facilitating CO_2_ transfer and fixation. In addition to the structural parameters of the aeration device, the ventilation conditions, including the gas flow rate and inlet CO_2_ concentration in the mixed gas, had significant effects on the bubble behavior and microalgal growth (Zhao et al., 2015), as shown in Figure 6. Figures 6A, B show the variation in the growth curve and pH with different inlet CO_2_ concentrations (3%, 5%, and 10%) in the microalgae photobioreactor with the optimal aeration device (gg, *δ* = 1 mm, *M* = 0 mm) and the gas flow rate was maintained at 70 mL/min. Therefore, it can be concluded that when the inlet CO_2_ concentration supplied was 5%, microalgae grew the fastest, and the maximum microalgae dry weight and productivity were achieved (Figure 6A). This was because an inlet CO_2_ concentration of 10% resulted in severe acidification of the suspension and the lowest pH (Figure 6B) owing to the higher CO_2_ dissolution rate, smaller bubble size, and longer bubble residence time, which completely inhibited microalgae growth (Chiu et al., 2008; Yoo et al., 2010). Moreover, a higher inlet CO_2_ concentration caused severe bubble-carrying, and fewer cells were present in the middle of the suspension, impeding microalgal growth (Ding et al., 2016). In contrast, when the inlet CO_2_ concentration was lower, the cells lacked CO_2_ supply, the pH was maintained at a high level, and the maximum pH reached 7.36 (Figure 6B).

Furthermore, the effects of gas flow rate (30, 70, and 140 mL/min) on microalgae growth in a microalgae photobioreactor with the optimal aeration device (gg, *δ* = 1 mm, *M* = 0 mm) were investigated when the inlet CO_2_ concentration supplied was 5% (Figures 6C, D). The best performance was achieved at the 70 mL/min gas flow rate. This was because a lower gas flow rate caused an insufficient CO_2_ supply, restricting microalgal growth (Figure 6C), causing less gas-liquid interface area, and the weaker interface oscillation hindered CO_2_ transfer. Therefore, the lowest biomass productivity of 0.15 g/(Ld) and highest pH of 7.4 was achieved at a 30 mL/min gas flow rate (Figure 6D). However, a larger gas flow rate (140 mL/min) induced more gas-liquid interface area and stronger interface oscillation, causing CO_2_ excess supply, impeding algal growth owing to the lowest pH and more acidic suspension (Chiswell et al., 1999; Vogel et al., 2010). In addition, a higher gas flow rate caused a higher bubble-carrying coefficient owing to the generation of more bubbles per unit time, which was unconducive to algal growth (Ding et al., 2016).

To sum up, the optimal performance was obtained in the bubble column photobioreactor with the novel aeration device (gg, *δ* = 1 mm, *M* = 0 mm) when inlet CO_2_ concentrations was 5%, gas flow rate was 70 mL/min, and the maximum dry weight and biomass productivity reached 1.53 g/L and 0.24 g/(Ld), respectively.

## 4 Conclusion

In the present study, the dynamic behaviors of bubble cutting in a photobioreactor with a novel aerator were investigated, and the effects of thickness, hydrophilicity, and arrangement of cutting slices on microalgal growth were investigated. The experimental results were as follows.

1) Bubble cutting decreased the bubble diameter by 27.97%, decreased bubble rising velocity by 46.88%, and prolonged the residence time by 84.55%, promoting CO_2_ mass transfer. The maximum dry weight and biomass productivity of microalgae improved by 6.99% and 33.33%, respectively, compared with those of the bioreactor without cutting units.
2) The bubbles were successfully cut when the slices were arranged in parallel, and the thickness and spacing of the cut slices were less than 3 mm.
3) Although the PTFE slice could prolong the bubble residence time by preventing daughter bubble departure, it adversely affected microalgal growth owing to bubble coalescence with subsequent bubbles.
4) The optimal photobioreactor performance was obtained in a bubble column photobioreactor with the novel aeration device (gg, *δ* = 1 mm, and *M* = 0 mm) when inlet CO_2_ concentrations was 5%, the gas flow rate was 70 mL/min, and the maximum dry weight and biomass productivity reached 1.53 g/L and 0.24 g/(Ld), respectively.

Overall, the novel aeration device with bubble cutting can greatly improve photobioreactor performance. However, it still has many limitations and potential challenges in large-scale applications. For example, the installation accuracy of cutting slices is difficult to ensure as the aeration area expands, which may affect the cutting success rate. Moreover, multi-layer cutting should also be considered to further improve CO_2_ dissolution efficiency.

## Data Availability

The original contributions presented in the study are included in the article/supplementary material, further inquiries can be directed to the corresponding author.

## Abbreviations

gg, pure glass; pp, pure PTFE; gp, half glass, half PTFE.
